# Water sources and composition of dissolved gases and bubbles in a saline high Arctic spring

**DOI:** 10.1371/journal.pone.0282877

**Published:** 2023-04-03

**Authors:** Dale T. Andersen, Christopher P. McKay, Wayne H. Pollard, Margarita M. Marinova

**Affiliations:** 1 Carl Sagan Center, SETI Institute, Mountain View, California, United States of America; 2 Space Science Division, NASA Ames Research Center, Moffett Field, California, United States of America; 3 Department of Geography, McGill University, Montreal, Quebec, Canada; Centro de Astrobiologia, SPAIN

## Abstract

We investigate the water sources for a perennial spring, “Little Black Pond,” located at Expedition Fiord, Axel Heiberg Island in the Canadian High Arctic based on dissolved gases. We measured the dissolved O_2_ in the likely sources Phantom Lake and Astro Lake and the composition of noble gases (^3^He/^4^He, ^4^He, Ne,^36^Ar, ^40^Ar, Kr, Xe), N_2_, O_2_, CO_2_, H_2_S, CH_4_, and tritium dissolved in the outflow water and bubbles emanating from the spring. The spring is associated with gypsum-anhydrite piercement structures and occurs in a region of thick, continuous permafrost (400–600 m). The water columns in Phantom and Astro lakes are uniform and saturated with O_2_. The high salinity of the water emanating from the spring, about twice sea water, affects the gas solubility. Oxygen in the water and bubbles is below the detection limit. The N_2_/Ar ratio in the bubbles and the salty water is 89.9 and 40, respectively, and the relative ratios of the noble gases, with the exception of Neon, are consistent with air dissolved in lake water mixed with air trapped in glacier bubbles as the source of the gases. The Ne/Ar ratio is ~62% of the air value. Our results indicate that about half (0.47±0.1) of the spring water derives from the lakes and the other half from subglacial melt. The tritium and helium results indicate that the groundwater residence time is over 70 years and could be thousands of years.

## Introduction

Perennial springs in regions of thick, continuous permafrost are uncommon, and understanding the source of water and the processes that determine the location and flow rates of these springs is of interest and has implications for hydrological processes on Mars. In this study, we focus on a single perennial spring, known as “Little Black Pond,” located at the base of Gypsum Hill, Expedition Fiord on Axel Heiberg Island in the Canadian Arctic Archipelago.

While uncommon, perennial springs in high latitudes have been reported in several locations including, Ellesmere Island [1, 2], Spitzbergen [3], and Axel Heiberg Island [4]. On Axel Heiberg Island, there are two active sets of perennial, saline springs that drain into Expedition Fiord: Colour Peak Springs and Gypsum Hill Springs [5–8]. Both sets of springs emerge from the base of gypsum-anhydrite piercement dome structures (diapirs).

**Fig 1:**
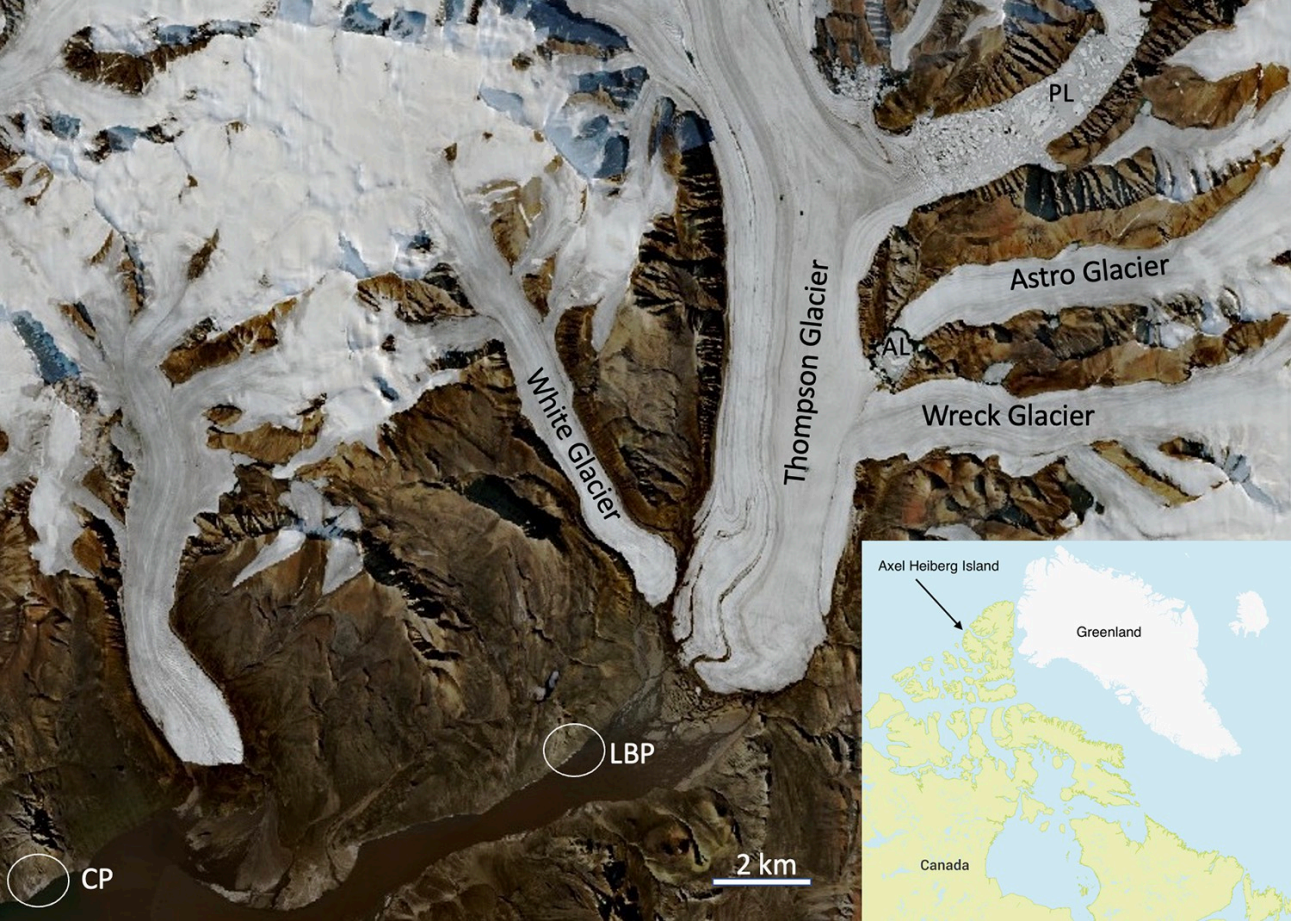
Context image showing the location of Little Black Pond (LBP) and Colour Peak Springs (CP), the glaciers and ice-dammed lakes: Astro Lake (AL) and Phantom Lake (PL). Image from Copernicus Sentinel-2 L2A 2020-07-11 processed with EO Browser. Inserts showing the location of Axel Heiberg Island and the location of the site on the island.

The study area on Axel Heiberg Island (Fig 1), including both Little Black Pond, part of the Gypsum Hill Springs, and Colour Springs, is located in the center of an extensive geological feature known as the Sverdrup Basin. This basin is 500 km wide and up to 13 km deep and extends 1300 km southwest from northern Ellesmere Island [9]. The Sverdrup Basin is filled with layers of Carboniferous to Paleogene strata [9]. The study area is within a 60-km-wide area, known as the wall-and-basin structure (WABS) province [10, 11]. Detailed geological analysis and maps of the WABS province are in Harrison and Jackson [10] and Jackson and Harrison [11]. The WABS province has bimodal fold trends and irregular regional anticlines spaced at <10 km intervals [10, 11] that are correlated with diapirs of superficially gypsified anhydrite crop from an autochthonous evaporite layer comprising halite overlain by thick anhydrite [11]. Diapirs are widespread on Axel Heiberg Island; forty-six diapirs of Carboniferous evaporites and associated mini basins are exposed on the island, as shown in Fig 1 of Harrison and Jackson [10].

The Colour Peak Springs are located on the south-facing slope of Colour Peak at an approximate elevation of 100 m a.s.l., emerging from the top of the slope along a line nearly 400 m long. These springs are grouped into three distinct topographically controlled areas with 20 or more vents discharging directly into Expedition Fiord three hundred meters down slope. The Gypsum Hill spring site consists of approximately 40 springs and seeps on the northeast side of Expedition River, discharging along a band nearly 300 m long and 30 m wide between 10–20 m a.s.l. [4]. Little Black Pond is located within the group of outlets at the Gypsum Hill spring location.

This region of the high arctic is heavily glaciated and has a mean air temperature of –15°C [4, 12]. Permafrost depth has not been measured directly at Expedition Fiord; however, a permafrost thickness > 400 m was documented in an exploration well at Mokka Fiord on the east side of Axel Heiberg Island, roughly 60 km from Expedition Fiord. Other exploration wells in the area reveal that permafrost is generally between 400–600 m thick [13]. Permafrost features include extensive polygonal ice wedge development in unconsolidated fluvial and colluvial deposits at lower elevations. The presence of thick, continuous permafrost in the Arctic Archipelago makes it difficult for groundwater to reach the surface or for surface waters to recharge deep aquifers [14]. These springs may have formed while submerged in the past when the marine limit was higher, as suggested for the springs in Spitzbergen by Haldorsen and Heim [3].

Permafrost springs have been suggested as models for water flow on Mars [7, 15, 16] and as sites with the possibility of supporting active ecosystems or preserving evidence of life [7, 17–20]. The discovery of relic springs [21] is of further interest in this regard.

Andersen et al. [7] developed a combined flow and thermal model of the subsurface flow associated with the springs at Expedition Fiord. Their data show the thermal and flow properties of the springs remain remarkably constant given the harsh climate and extreme seasonal variations in air temperature, and they noted that the thermal properties of the springs are controlled by the local geothermal gradient. They postulated that the groundwater is derived from local glacially dammed alpine lakes and/or subglacial discharge infiltrating the sedimentary units associated with the numerous anhydrite piercement structures prevalent in the area. Phantom Lake and Astro Lake, both proglacial lakes abutting the Thompson Glacier, connect to these diapirs and provide the most likely sources of lake water for the springs. As pointed out by Haldorsen and Heim [3], subglacial melt may also provide a means for the deep circulation of groundwater beneath ice sheets and glaciers. For example, the White Glacier is a nearby multithermal alpine glacier with permafrost at the lobes and melting bottom temperatures occurring above the equilibrium line [22]. The larger Thompson glacier is also a likely source of subglacial melt entering the diapirs that are around and below that glacier.

In several locations, as the spring water emerges from the ground, it forms pools, and gas bubbles stream upward toward the surface, indicating that the water is saturated with gases. The nature of these gases must reflect the source of the water and processes in the subsurface. The water in Phantom and Astro Lake is likely to have dissolved gases in equilibrium with the atmosphere, while subglacial melting may carry gases set by the bubbles in the glacial ice–not in equilibrium with the atmosphere. The differing patterns of gases in the two sources can, in principle, be used as a signature of the mix that makes up the spring water. In this study, we report on the concentration of the gases dissolved in the spring water and the composition of the bubbles emerging from the spring outflow at Gypsum Hill Spring.

## Methods

**Fig 2:**
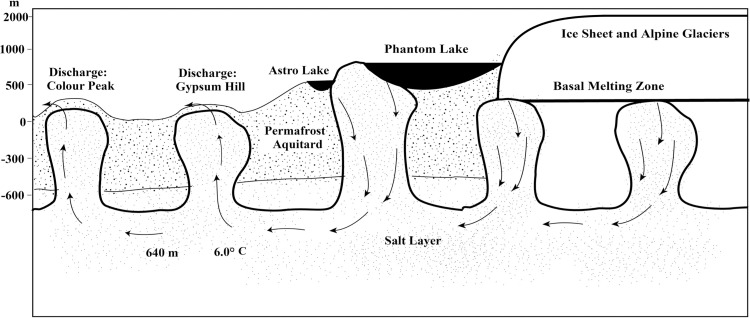
Schematic diagram depicting the sources of gas and water showing the contribution of lakes and subglacial melt and the flow of subsurface water through the salt strata associated with the diapirs. Modified from Andersen et al. [7].

**Fig 3:**
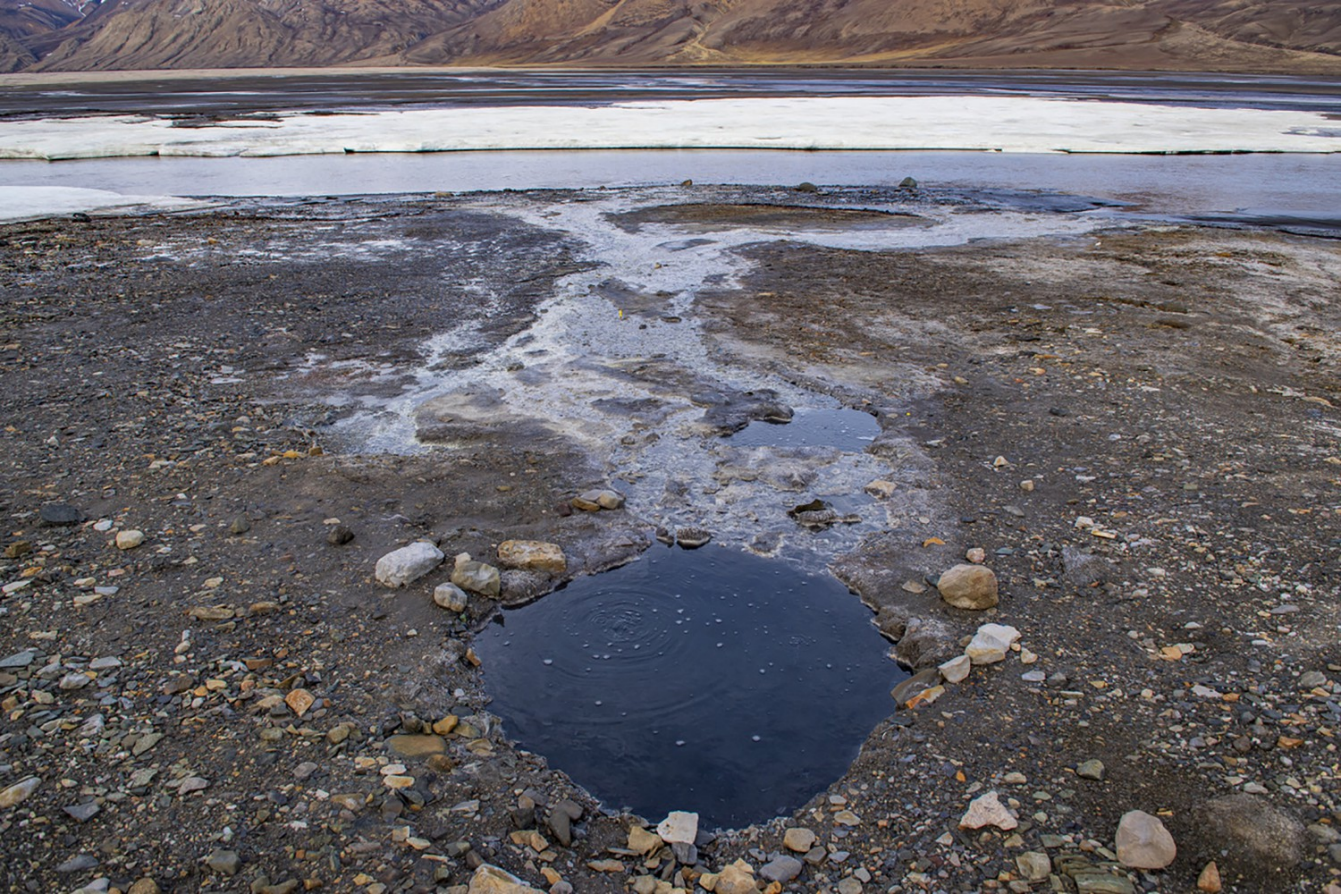
Close-up of Little Black Pond in the Gypsum Hill springs with the Expedition River in the background. The pond is about 1 m in diameter.

**Fig 4:**
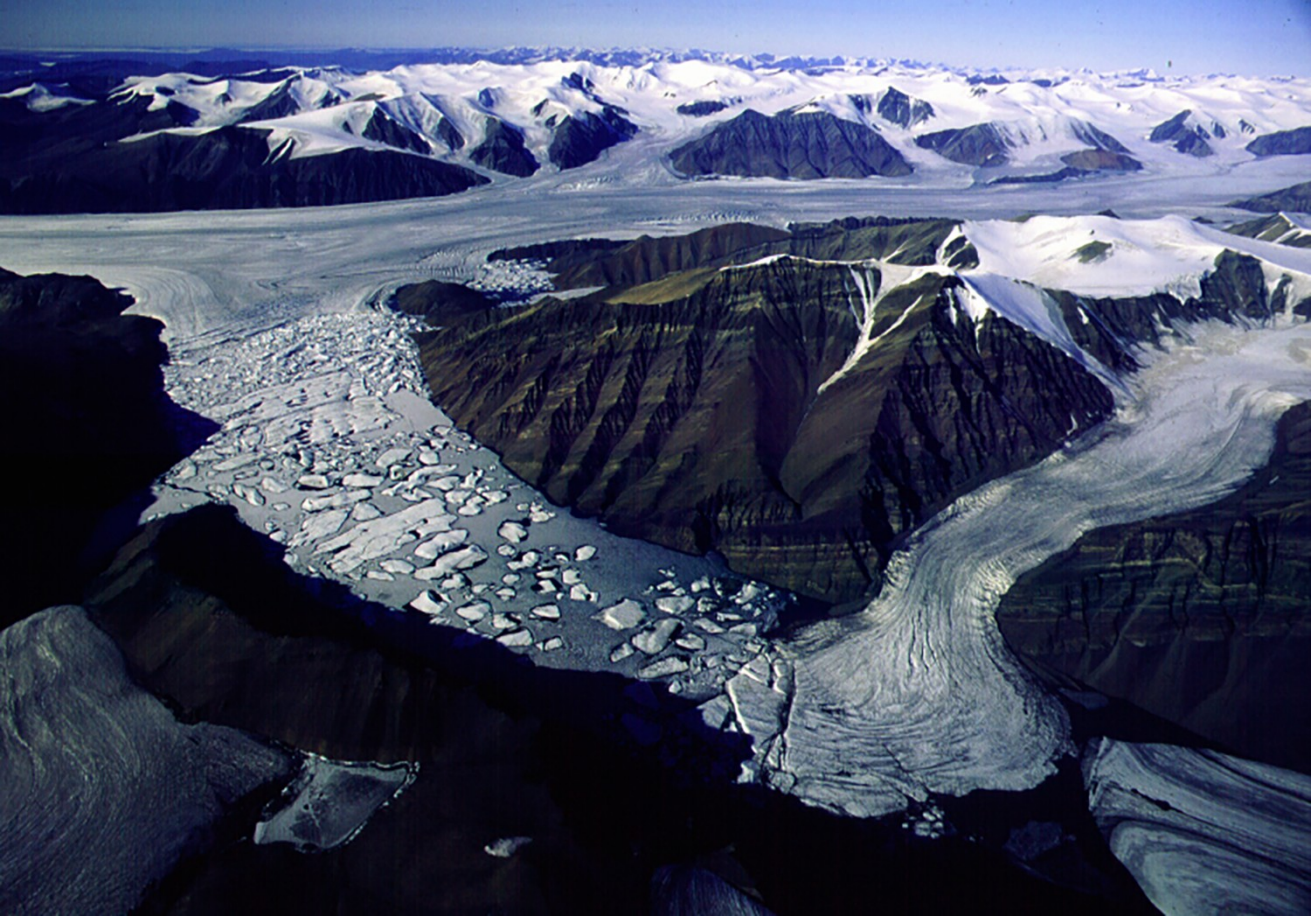
Aerial image of Phantom Lake looking toward the Thompson Glacier, May 2003. The lake is 4 km long.

Samples collected for this study were obtained at the base of Gypsum Hill in a pool-type outlet known informally as “Little Black Pond” (N79.40409° W90.73309°, elevation ~19m). The pool is approximately 1 meter in diameter and is filled with black, coarse-grained sediment. Fig 1 shows the local site and its location on Axel Heiberg Island in the Arctic. Fig 2 shows a diagram of the source and underground flow of the springs based on Pollard et al. [5] and Andersen et al. [7]. The 6°C water temperature and flow rate of ~ 200 mL/s have remained constant over the 5 years of measurement activity [7]. Fig 3 shows an image of Little Black Pond with the Expedition River flood plain seen in the background. Fig 4 shows an aerial image of Phantom Lake.

Two techniques were used to collect samples. Water samples for dissolved noble gases, including isotopic composition, were collected in 0.95 cm (3/8 inch) diameter Cu tubes clamped at both ends. These samples were delivered to Lawrence Livermore National Lab (LLNL) for analysis. Borosilicate serum vials, 60 cm^3^, were flushed and inverted in the brine to obtain both water samples and bubbles emerging from the spring. *In situ* depth, temperature and dissolved O_2,_ measurements were obtained in Phantom and Astro Lakes using a YSI multiparameter sonde 6600 (YSI Incorporated, Yellow Springs, Ohio, USA) and with an Idronaut, Ocean Seven 316 multi-parameter probe (Brugherio (MB), Italy). A YSI Model 95 (YSI Incorporated, Yellow Springs, Ohio, USA) handheld dissolved oxygen and temperature system with automatic temperature and salinity compensation was used to determine the presence or absence of oxygen in Little Black Pond.

Samples were analyzed for noble gases (^3^He/^4^He, ^4^He, Ne, Ar, Kr, Xe) at the LLNL using standard isotope dilution techniques using a VG5400 noble gas mass spectrometer following methods outlined by [23]. ^36^Ar, ^40^Ar, N_2_, O_2_, CO_2_, and CH_4_ were measured at NASA Ames Research Center using a SRS model RGA-200 quadrupole mass spectrometer (Stanford Research Systems, Sunnyvale, CA) and a Varian 5700 GC equipped with a Saturn IV mass spectrometer for the detection system. For gas analysis, the sample was introduced into the vacuum system connected to the RGA-200 spectrometer through a leak valve. Air was used as the primary standard gas for concentration and ratio measurements. For water samples, a headspace of 5 mL was first produced using He as described by Risgaard-Petersen and Rysgaard [24] and Blicher-Mathiesen et al. [25]. For the measurement of dissolved He the headspace was produced with air. Total H_2_S was sampled and analyzed according to [26]. Direct and enriched tritium analyses of local meteoric water and the spring water were conducted at the Environmental Isotope Lab, Dept. Of Earth Sciences, University of Waterloo, Waterloo, Ontario, Canada. Spring discharge samples were analyzed for tritium by the electrolytic enrichment method, which has a detection limit of 0.8 ± 0.8 T.U. at 2 sigma while other locally collected samples were measured using the direct count method, which has a detection limit of 6.0 ± 6.0 T.U. at 2 sigma.

The solubilities of He, Kr, and Xe were calculated with the method of Benson and Krause [27] and corrected for the presence of salt using the noble gas salting coefficients in Table 3 of Smith and Kennedy [28]. For Ne, Ar, and N_2_, the solubility and salting out effect were determined from the equations and coefficients from Hamme and Emerson [29].

The analytical precision for the noble gas measurements at LLNL was 1%. The standard deviation for four replicates were: ^3^He/^4^He (4.3%), ^4^He (14%), Ne (14%), Ar (4.4%), Kr (1.4%), Xe (3.0%). The measurement uncertainty in absolute gas abundance determined with the RGA-based analyses was 10% with three replicate samples, while the uncertainty in the ratio, N_2_/Ar, was 3%, and for ^40^Ar/^36^Ar, was < 1%.

All research and sampling reported in this paper were conducted out of the McGill Arctic Research Station, operated by McGill University and directed by co-author WHP. A Scientific Research License from the Nunavut Research Institute (NRI) as well as other required permits and environmental assessments were obtained prior to this study by co-author WHP.

## Results

Gas concentrations in the bubbles are presented in Table 1, and dissolved gases in the spring water in Table 2. The gas in the bubbles is composed primarily of N_2_ with relative concentrations of Ar, Kr, and Xe that are almost identical to air (Kr/Ar and Xe/Ar are 1.01 and 1.02 times their air values respectively). The ratio of ^40^Ar/^36^Ar in the bubbles is indistinguishable from the atmospheric value, 298.6, and is listed in Table 1. O_2_ is undetectable, and ^4^He is enriched by a factor of about 500. Ne is depleted, the Ne/Ar ratio is ~62% of the air value. CO_2_ and CH_4_ are present at low levels. Dissolved H_2_S was determined to be 30 mg/L. Tritium content measured in local meteoric sources (lakes and glacial runoff) ranged from 13–15 ± 6 T.U. while our measured value for Little Black Pond was below the detection limit, < 0.8 ± 0.8 T.U. The tritium in Colour Peak Springs was also below the detection limit, <0.8 ± 0.8 T.U.

**Table 1:** Summary of data for gas in bubbles.

Gas	Gas Bubbles Volume Mixing Ratio	Standard Dry Air Volume Mixing Ratio
Ar	11,100 ppm	9,340 ppm
Ne	13.5 ppm	18.18 ppm
Kr	1.37 ppm	1.14 ppm
Xe	0.105 ppm	0.087 ppm
^4^He	*2810 ppm*	5.24 ppm
N_2_	*0*.989*	0.781
O_2_/N_2_	<0.001	0.27
N_2_/Ar	89.9*	83.6
^40^Ar/^36^Ar	298	298.6
CO_2_	350 ppm**	417 ppm
CH_4_	2600 ppm**	1–2 ppm

*Measured with RGA200, NASA ARC, and calibrated by Ar + N_2_ = 1

**Measured with Varian GCMS

**Table 2:** Summary of data for gas dissolved in spring water.

	Gypsum Hill Spring*	Standard Dissolved Air @ 0° C
Ar	3.36 × 10^−4^ cm^3^ STP/g	5.0 × 10^−4^ cm^3^ STP/g
Ne	1.02 × 10^-7^cm^3^ STP/g	2.26 × 10^−7^ cm^3^ STP/g
Kr	8.8 × 10^−8^ cm^3^ STP/g	1.26 × 10^−7^ cm^3^ STP/g
Xe	1.18 × 10^−8^ cm^3^ STP/g	1.97 × 10^−8^ cm^3^ STP/g
N_2_/Ar	40**	35.5
^4^He	1.65 × 10^-5^cm^3^ STP/g	
^3^He/^4^He	8.83 × 10^−8^	
Tritium	< 0.8 ± 0.8 T.U.	13–15 ± 6 T.U.***
H_2_S	30 mg/L	

* Per unit gram of saline spring water

** Measured with RGA200, NASA ARC, and normalized to Ar + N_2_ = 1

*** Samples taken from local glacial meltwater streams and from small lakes

**Fig 5:**
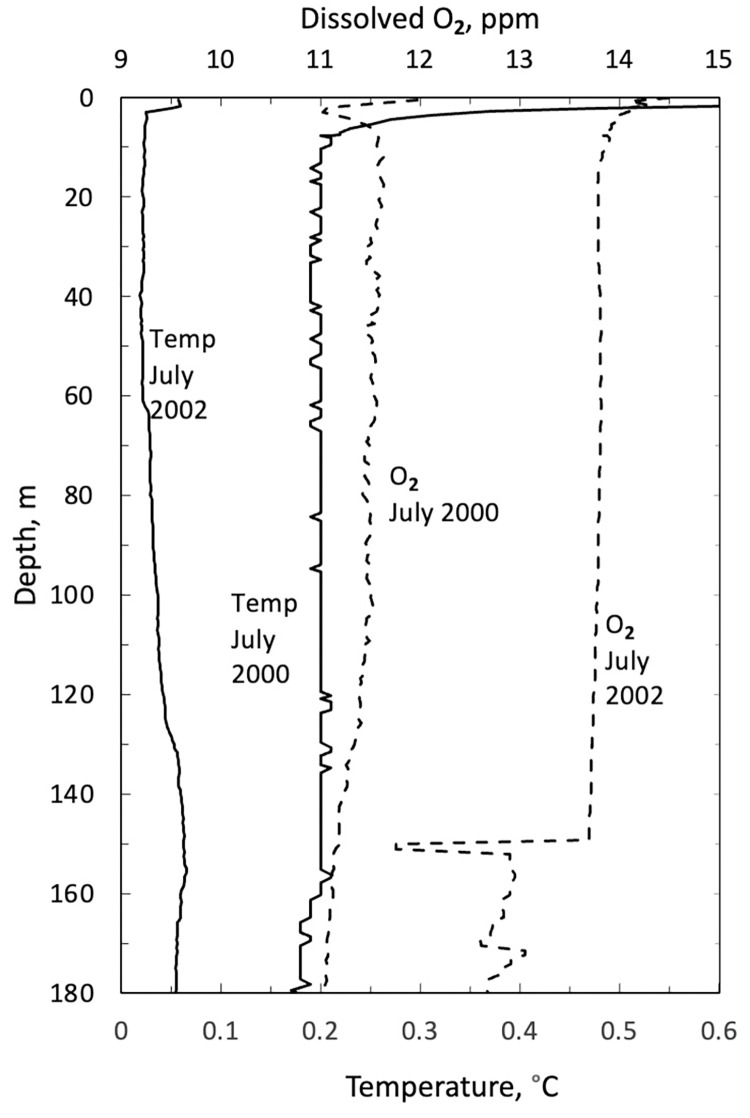
Temperature and dissolved oxygen in Phantom Lake in July of 2000 and 2002.

**Fig 6:**
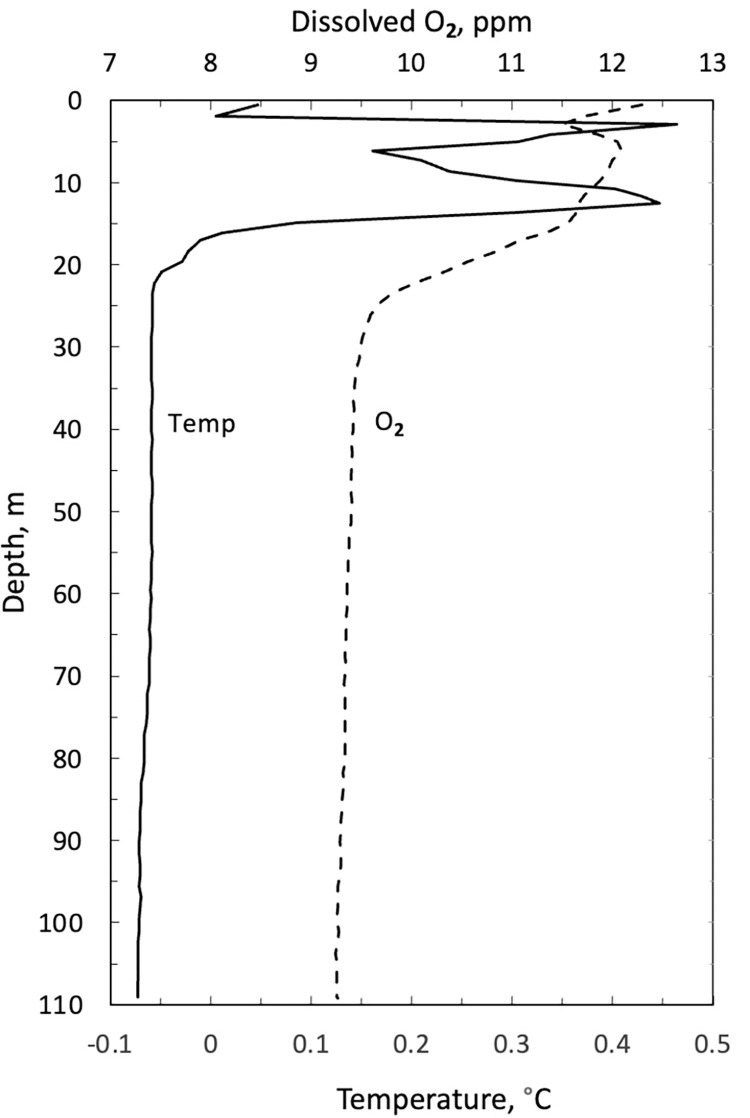
Profile of temperature and dissolved oxygen in Astro Lake on 7 July 2002.

The water columns of Phantom Lake and Astro Lake were determined to be well-mixed, isothermal, and oxygenated. Broadly, the water temperature is within ±0.5°C of freezing, and the O_2_ levels are ~ 9–14 ppm (~80–100% of atmospheric equilibrium) throughout the water column. Plots of the temperature and dissolved oxygen profiles for Phantom are shown in Fig 5 for July 2000 and 2002. The profile for Astro Lake for July 2000 is shown in Fig 6. Dissolved oxygen was not detected within the water emerging from Little Black Pond.

## Discussion

Visual observations of Little Black Pond suggest that the large, emergent gas bubbles may be in equilibrium with the salty water flowing from the spring. We have determined the extent of this by comparing the measured partial pressure of each gas in the bubbles in the spring water to the value expected for equilibrium with the measured dissolved gas in the spring water. Conceptually, we consider a parcel of water at high hydrostatic pressure deep below the surface when all gases are in solution. As the water nears the surface, the hydrostatic pressure drops, and bubbles form. The total gas content is partitioned into the bubble volume and the spring water. In equilibrium, the partial pressure of gas, *i*, in the bubble, *P*_*i*_, is equal to the partial pressure of that gas determined by its mixing ratio in the liquid, *P*^*s*^_*i*_. The values of *P*_*i*_ are determined by the measured mixing ratios of each gas in the bubbles and the requirement that the total gas pressure in the bubble sums to atmospheric pressure [30]. The values of *P*^*s*^_*i*_ are determined from the measured mixing ratios and the solubility coefficients

$$P^{s}{}_{i}=\rho X_{i}/\beta_{i}(T,S)$$ (1)

where, *ρ* is the density of the saline spring water (kg/liter), *X*_*i*_, is the dissolved gas content (cm^3^ STP per kg of spring water) measured in the spring water, *β*_*i*_*(T*,*S)* is the Bunsen coefficient (in cm^3^ STP/atm per liter water) which is defined as the volume of gas measured at STP that is dissolved per liter of solution, when the partial pressure of the gas is 1 atm [27] at the temperature, *T*, of the spring water (6°C) and salinity, *S*.

**Fig 7:**
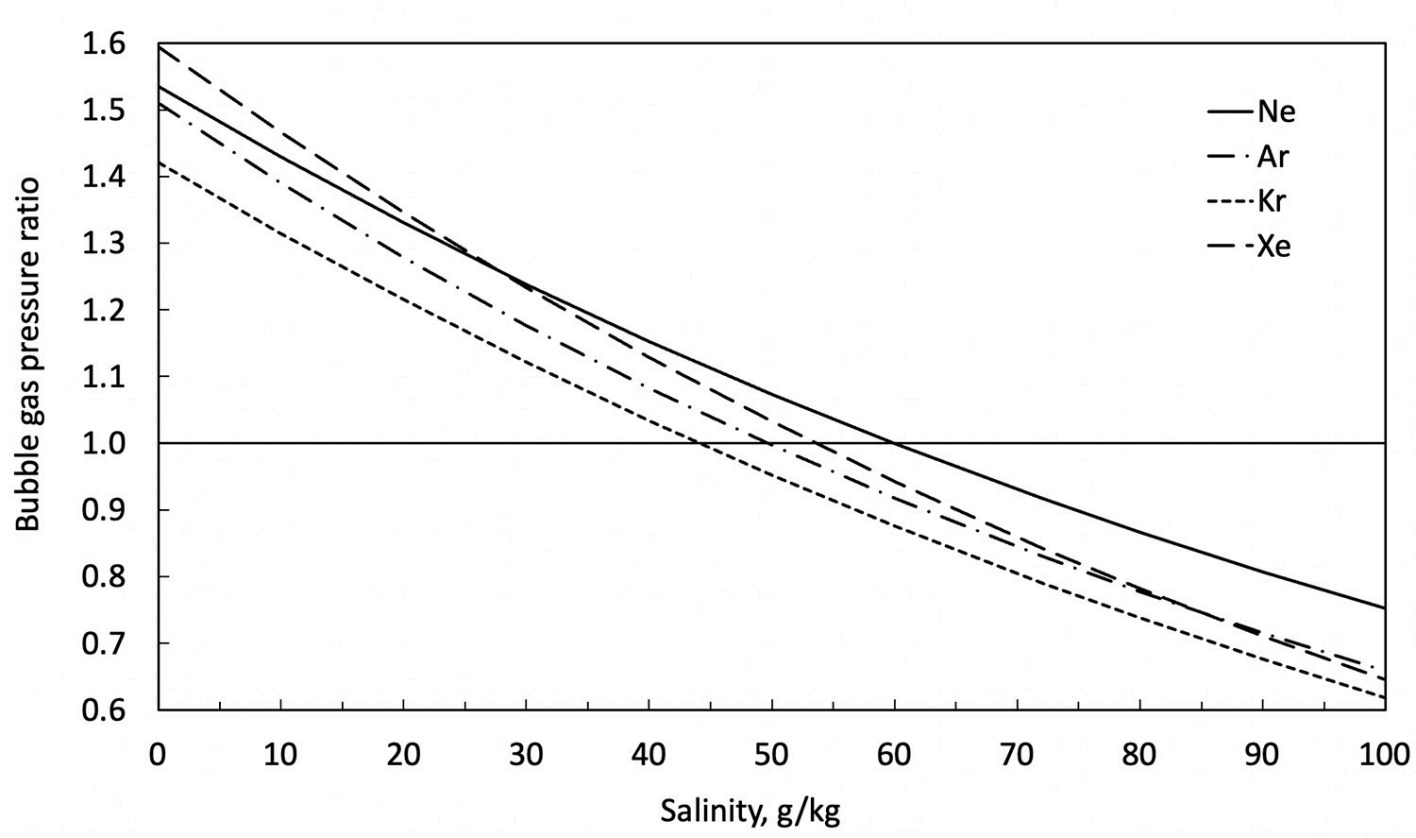
The bubble gas pressure ratio is the measured partial pressure in the bubbles for each gas divided by the computed partial pressure in the bubble based on the measured salt concentration in the spring water. This is plotted for each gas as a function of salinity. As the salinity increases, the solubility of gases decreases. The bubble gas pressure ratios are ~0.8 at the measured values for salinities near the level in the springs (72 g/kg), indicating that the gas in the bubbles is 20% lower than the equilibrium–the gases do not fully achieve equilibrium with the dissolved gases.

The ratio of *P*_*i*_/*P*^*s*^_*i*_ for each gas dissolved in the spring water is plotted in Fig 7 as a function of salinity. For salinities near 72 g/kg, the salinity of the spring water reported by Pollard et al. [5], the measured values divided by the predicted values are all close to unity but systematically low by ~ 20%, suggesting that the bubbles are not quite in equilibrium with the water. The spring water has mostly outgassed to the bubbles but not completely. Approximate equilibrium is expected since the spring water is flowing slowly through loose, coarse-grained material at the bottom of the pool, which provides surface area that helps establish equilibrium. Based on this analysis of the equilibrium, when we compute the mass balance of the gases, we use both the direct measurements of the dissolved gases in the water and the direct measurements of the partial pressure of the gases in the bubbles. But we also consider as an alternative case the assumption that the partial pressure in the bubbles is set by equilibrium with the brine. Thus, for our final mass balance we compare to these two reference values.

The lack of O_2_ implies it is consumed by chemical and biological processes during the transit of the water below ground. The low levels of CO_2_, H_2_S, and CH_4_ compared to the expected level of O_2_ in the source water (~ 20%) implies that most of the consumption of O_2_ does not result in gas formation but a soluble compound, such as sulfate due to reaction with sulfides. This is consistent with the high levels of sulfate (3700 mg/L) reported in the spring water by Pollard et al. [5]. The CO_2_ and CH_4_ are likely to reflect reactions with ancient sedimentary organic material as the water moves through the salt diapir, which may introduce a reservoir effect making radiocarbon dating of the spring problematic [31]. The H_2_S is likely to be due to microbial reduction by the reaction of organic matter with sulfate.

Groundwater can accumulate gases produced in geological layers by the radioactive decay of long-lived elements. In particular, ^4^He is produced by the decay of U and Th, and ^40^Ar is produced by the decay of ^40^K. Our results (Table 2) indicate that the ^40^Ar in the spring is consistent with an atmospheric source and a negligible geogenic source, while ^4^He in the bubbles is greatly in excess of atmospheric values (~500 times, Table 1), indicating an important geogenic source.

The ^3^He/^4^He in the spring water, 8.83 × 10^−8^, is much smaller than the atmospheric value (1.340 ± 0.006 × 10^−6^ [32] consistent with ^4^He resulting from geologic sources. The tritium in the spring water is much less than found in surface waters in the vicinity of the spring. The age of the water cannot be accurately determined from our measurements. However both the ^4^He and tritium are consistent with a long residence time [33]. Tritium in the water of Little Black Pond is quite low or undetectable (Table 2). In Colour Peak spring, tritium was <0.8 ± 0.8 T.U. The tritium in both springs is much smaller than the local surface waters (13–15 ± 6.0 T.U., Table 2), indicating the spring water is pre-bomb (>70 years). The accumulation of ^4^He in the spring water bubbles to approximately 500 times atmospheric levels (Table 1), indicates a possibly much older age for the water—it is uncertain but could be up to many thousands of years

The two sources of water suggested for the springs at Expedition Fiord are lake water and glacial ice [7]. The air gases (N_2_ and noble gases) could be derived from air dissolved in surface meltwater (e.g., Phantom Lake and Astro Lake) that enters the subsurface by infiltrating faults in the piercement structures described by Pollard et al. [5] and as shown in Fig 2. Both lakes have water temperatures of ~0°C, and measurements of dissolved O_2_ show that both are close to atmospheric air equilibrium throughout their water columns (Figs 5 and 6). An alternate source is gas inclusions trapped in glacial ice that are discharged into the subsurface during basal melting. If the basal meltwater is isolated from the atmosphere, the composition of the gas will be maintained since the gases in the glacial ice will be forced into solution. If the basal meltwater is not isolated from the atmosphere, it will come into air equilibrium and be indistinguishable from lake water. Presumably, melting and gas injection occur at the base of glaciers that are overriding the regional piercement structures previously discussed.

The N_2_/Ar ratio can be used as an indicator of the source of water and gas in permafrost because of the distinct difference in this ratio between air trapped in bubbles in glacier ice and gases dissolved in water [34]. With O_2_ removed, only N_2_ and Ar are significant in the mass balance of gas in the spring.

The mass balance equation for each gas species in terms of a unit mass of water can be expressed as the volume of gas (cm^3^ STP) entering the spring from the air-saturated fresh lake water plus the volume of gas carried into the spring from bubbles in the glacier ice. The volume of gas leaving the spring at the outlet is the sum of the gas partitioned between the salty spring water and bubbles. The mass balance for any gas in terms of volume per unit mass of fresh water is thus:

$$W\;P^{l}{}_{i}\;\beta_{i}(0,0)/\rho_{o}+(1‐W)V\;P^{g}{}_{i}=[X_{i}+Vb\;P_{i}]\rho_{o}/\rho$$ (2)

where *W* is the mass fraction of the incoming water from the lake, *1-W* is therefore, the mass fraction due to glacier meltwater. As discussed above in Eq (1), *β*_*i*_*(T*,*S)* is the Bunsen coefficient (in cm^3^ STP/atm per liter solution) and is defined as the volume of gas measured at STP that is dissolved per liter of solution when the partial pressure of the gas is 1 atm [24], *β*_*i*_*(*0,0*)* refers to the coefficient for the lake water at 0°C with zero salinity, *β*_*i*_*(T*,*S)* refers to the coefficient for the spring water at the corresponding temperature (6°C) and salinity, *S*. *P*^*l*^_*i*_ is the atmospheric partial pressure (in atms) of the gas above the air-saturated lake water which is the normal sea level atmospheric partial pressures reduced by altitude, *ρ*_*o*_ is the density of fresh water. *V* is the volume of gas at standard temperature and pressure (cm^3^ STP per kg) in glacial ice per kg of ice, *P*^*g*^_*i*_ is the atmospheric partial pressure (in atms) of the gas above the glacier, which is the normal sea level atmospheric partial pressures reduced by elevation. The gas content of the glacial ice depends both on the altitude at the site of ice accumulation and on the pore volume in the firn. Martinerie et al. [35] report on values of *V* from 60 to 140 cm^3^ STP per kg as a function of the elevation of the glacier. *Vb* is the volume of gas bubbles in the spring water per kg of spring water, *P*_*i*_ is the partial pressure of the gas in units of atms in the bubbles at the spring outlet, which we determine directly from the measured mixing ratios of the bubbles (and as discussed above in the analysis of the equilibrium at high salinity), *ρ* is the density of the spring water (liters/kg), *X*_*i*_ is the dissolved gas content (cm^3^ STP per kg) measured in the spring water. The term *ρ*_*o/*_*ρ* corrects for the mass added to the solution by the salt so that right-hand side of Eq 2 is the gas per unit mass of fresh water.

Eq 2 represents one equation for N_2_ (_*i*_ = N) and another for Ar (_*i*_ = A). If we specify the value of *V* and then use the data for N_2_ and Ar in the spring, we can use Eq 2 to determine *W* and *Vb*. Note that the volume of bubbles, *Vb*, cannot be measured accurately in the field and is therefore treated as a parameter to be determined from the mass balance. We set *V* = 100 cm^3^ STP per kg [35]. We assume that the atmospheric pressure above the lake water, at the glacier, and at the spring outlet is one atmosphere. The glacial gas inclusions are assumed to have exactly air composition in N_2_ and Ar. We note that there is fractionation of Ar to N_2_ in glacial bubbles of order 1% [36], which is small compared to the fractionation in the spring.

Solving Eq 2 for *P*_*i*_ gives

$$P_{i}=\{[W\;P^{l}{}_{i}\;\beta_{i}(0,0)/\rho_{o}+(1‐W)V\;P^{g}{}_{i}]\rho /\rho_{o}‐X_{i}\}/Vb$$ (3)

which can be used to compute the ratio of N_2_/Ar in the bubbles,

$$N_{2}/Ar=P_{N}/P_{A}$$

$$=\{[W\;P^{l}{}_{N}\;\beta_{N}(0,0)/\rho_{o}+(1‐W)V\;P^{g}{}_{N}]\rho /\rho_{o}‐X_{N}\}/\{[W\;P^{l}{}_{A}\;\beta_{A}(0,0)/\rho_{o}+(1‐W)V\;P^{g}{}_{A}]\rho /\rho_{o}‐X_{A}\}$$ (4)

Computing N_2_/Ar as a ratio has several advantages. First, the expression for the ratio, Eq 4, can be solved for *W* graphically by comparing to the measured values of the N_2_/Ar in the bubbles of the spring. Secondly, the ratio of N_2_/Ar is more accurately determined than the individual concentrations because common systematic errors are canceled out. An example of this is seen in Table 3, where the partial pressure of the bubbles determined by assuming the gases are in equilibrium (*P*^*s*^_*N*_ and *P*^*s*^_*A*_) are 25% larger than the measured partial pressure in the bubbles (*P*_*N*_ and *P*_*A*_). But the ratio of N_2_/Ar computed with these values is within 2% of the value measured directly in the bubbles. In addition, N_2_ and Ar are the only major gases, and they determine the volume of bubbles in the springs. Finally, the difference in solubility of N_2_ and Ar (Table 3) is more than a factor of two, resulting in a contrast in the ratio between air and gas dissolved in air-saturated water that is readily measured.

**Table 3:** Listing of the numerical values used in, or obtained from, the solution of Eq 4.

*W*	0.47 ± 0.01			
*Vb*	40 cm^3^/kg spring water			
*V*	100 cm^3^/kg ice			
*ρ*	1.072 kg/L			
*ρ*_*o*_	1.000 kg/L			
**Gas variable**	**N** _ **2** _	**Ar**	**Kr**	**Xe**
*P*^*l*^_*i*_ *= P*^*g*^_*i*_ atmospheric value	0.781 atm	0.00934 atm	1.14×10^−6^ atm	8.70×10^−8^ atm
*P*_*i*_ measured bubble value	0.989 atm	0.011 atm: gives ratio N_2_/Ar = 89.9	1.37×10^−6^ atm	1.05×10^−7^ atm: gives ratio Kr/Xe = 13.0
*β*_*i*_*(*0,0) solubility coefficient	23.86 cm^3^ STP L^-1^ atm^-1^	53.52 cm^3^ STP L^-1^ atm^-1^	110.92 cm^3^ STP L^-1^ atm^-1^	225.88 cm^3^ STP L^-1^ atm^-1^
*β*_*i*_*(T*,*S)* solubility coefficient	11.68 cm^3^ STP L^-1^ atm^-1^	26.91 cm^3^ STP L^-1^ atm^-1^	54.51 cm^3^ STP L^-1^ atm^-1^	100.88 cm^3^ STP L^-1^ atm^-1^
X_i_ Measured dissolved gas content	13.4 cm^3^ STP/kg	0.336 cm^3^ STP/kg	8.80×10^−5^ cm^3^ STP/kg	1.18×10^−5^ cm^3^ STP/kg
P^s^_i_ = ρ X_i_/β_i_(T,S) partial pressure calculated from dissolved gas content	1.23 atm	0.0134 atm: gives ratio N_2_/Ar = 91.8	1.73×10^−6^ atm	1.25×10^−7^ atm: gives ratio Kr/Xe = 13.8

**Fig 8:**
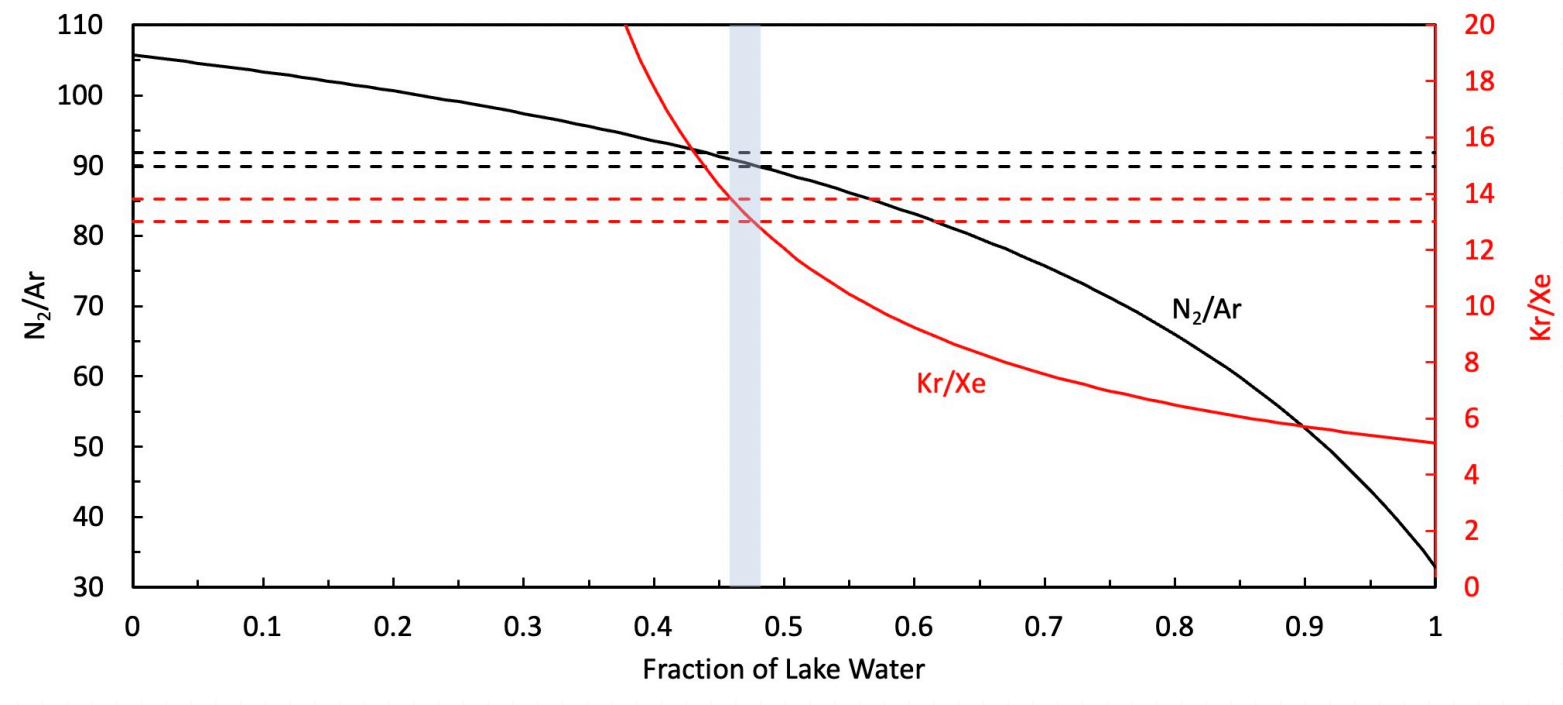
The calculated ratio of N_2_ to Ar in the bubbles in the spring as a function of the fraction of the source water that comes from air-saturated fresh water lakes. The other source is gas trapped in glacier ice carried into the springs. The dashed lines show the values of N_2_/Ar based on measured gas values in the spring water and the bubbles (lower line). The upper line shows the ratio for and the measured gas values in the spring water and the assumption of equilibrium in the bubbles in the spring. The uncertainty in the N_2_/Ar measurements is ±3%, comparable to the spacing between the two dashed lines (2%). The red line and the right-hand scale show the corresponding results for the ratio of Kr/Xe. The spacing between the red dashed lines is larger, ~ 6%. For both gas, ratios the fraction of liquid water that fits the data is 0.47±0.01, shown by the blue-shaded region.

In Fig 8, we show the calculated N_2_/Ar ratio in the spring bubbles corrected for the effect of salinity if the source water was only glacial ice (left end of Fig 8) and if the source water was only air-saturated water (right end of Fig 8) and values in between. The measured value is determined as the ratio of the measured partial pressures, *P*_*i*,_ of N_2_ and Ar, 89.9, or computed from the ratio of the *P*^*s*^_*i*_ (Eq 1: *P*^*s*^_*i*_ *= ρ X*_*i*_*/β*_*i*_*(T*,*S)*) in the spring water, which gives a value of 91.8. This difference of ~2% is comparable to the measurement error in the ratio (~3%). As discussed above, the ratios are similar because the bubbles are almost but not entirely in equilibrium with the salty solution, and other systematic errors cancel. Table 3 lists the parameters used in the calculations and the value of *Vb* (40 cm^3^/kg of spring water) that results from the calculation.

Fig 8 also shows the ratio Kr/Xe computed with the method of Eq 4. As discussed above, using the ratio of two gases improves the computation by minimizing systematic errors, such as the unknown volume of the bubbles in the spring and any errors in the correction for salinity. The shape of the curve for Kr/Xe is quite different from that for N_2_/Ar, presumably due to the difference in concentration and the very high solubility of Xe. As seen in Fig 8, the Kr/Xe results are in very good agreement with the N_2_/Ar results–both indicating that the fraction of lake water is 0.47±0.01. The agreement between the two independent gas ratios is so good that it motivates a numerical check that the results are not fixed by some computational error. If the solubility of Xe is reduced in the computation by just 10% or if the measured abundance of Xe in the spring water is reduced by 10%, the results are discordant—with the best fit for Kr/Xe indicating a fraction of lake water of 0.6.

Fig 8 shows that the gas balance is consistent with the source gas being a mix of half air-saturated lake water and half air derived from glacier bubbles. The volume of the bubbles in the spring water, *Vb*, is 40 cm^3^/kg of spring water (~ 4% of the volume of the water) at the surface. This is compared to 13.7 cm^3^/kg of gas dissolved in the spring water–the majority of the gas is coming out in the bubbles. Thus, the upwelling spring water should begin to form bubbles well below the surface.

As seen in Table 1, N_2_/Ar in the spring bubbles (~ 89.9) is close to the N_2_/Ar in air (83.6). But this does not indicate that the source of all gases is just bubbles of air. If an excess volume of air is forced into water that initially has no dissolved gases (such as melted pure ice), the bubbles that form will have an N_2_/Ar ratio greater than that of air because more Ar will be held in the water than N_2_. How much greater depends on the ratio of the volume of air to the volume of water. For our spring, the case of zero lake water–gas only from air composition bubbles added to melted ice–the predicted N_2_/Ar in the bubbles in the spring is 105, as seen in Fig 8, not 89.9. However, if the volume of the glacier gas added to the system, currently at 100 cm^3^ STP per kg, is increased to 270 cm^3^ STP per kg, then the predicted N_2_/Ar in the bubbles would equal the measured value of 89.9 for zero lake water. However, this value of the glacial bubbles exceeds reported limits, and the predicted volume of bubbles in the springs is 1/5 of the volume of water–much more than is observed. More importantly, the Kr/Xe values (in Fig 8) would still be inconsistent with the data for zero lake water. In summary, the N_2_/Ar data as well as the Kr/Xe concentrations, are all consistent with a glacier bubble volume of ~ 100 cm^3^ STP per kg and a fraction of lake water of ~ 0.5, with the N_2_/Ar providing the most accurate method for computing the value of the fraction of lake water for a given bubble volume and computing the volume of bubbles in the spring.

To understand the sensitivity of the result to the assumed value of the glacial gas content, *V*, we consider cases for *V* = 90 and 110 cm^3^ STP per kg ice. The value of *W* determined changes from 0.47 for the case of *V* = 100 cm^3^ STP per kg ice to 0.49 for *V* = 90 cm^3^ STP per kg ice and 0.46 for *V* = 110 cm^3^ STP per kg ice.

The relative ratios of the noble gases can also trace sources of water and gas in permafrost [37–39]. Other than neon, the ratios of noble gases in the spring are also consistent with an approximately equal mix of air-saturated water and air released from glacier bubbles. The depletion of neon by 60% of its air value is puzzling, especially because glacial sources of noble gases are expected to produce excess Ne, and in fact, this has been proposed as part of the noble gas signature of glacier ice [40]. Studies of the fraction of noble gases in the Antarctic Plateau do indicate a loss of Ne from the firn ice as it hardens with depth, but the loss is less than 1% [41]. It is noteworthy that others have also reported significant Ne depletion in glacial meltwater. Niu et al. [38] reported on gas in glacial meltwater in Greenland, and of the 13 samples studied, nine were classified as having relative depletions of Ne with respect to Ar, Kr, and/or Xe. A similar study [39] at the Athabasca Glacier also had five out of eight samples with deletion of Ne with respect to one of the heavier noble gases.

**Fig 9:**
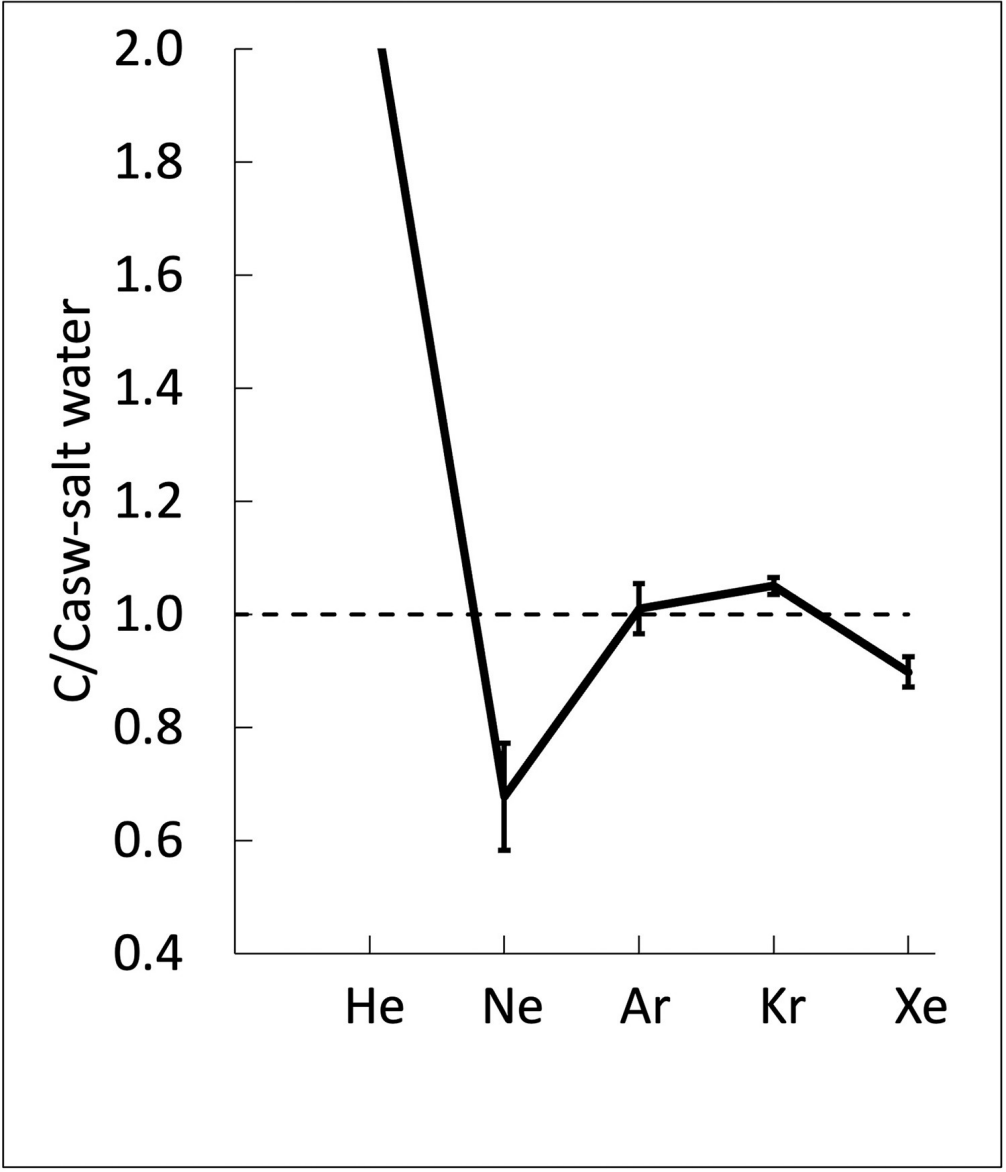
Noble case ratios in the springs normalized to air-saturated values for the saline spring water. The scale does not reach the level of He, which is hundreds of times higher in the spring bubbles than air levels due to He accumulation in the groundwater. Error bars are the standard deviation for four replicates.

Fig 9 shows the pattern of the noble gases in the spring water compared to air-saturated values for the brine plotted in the format used by Niu et al. [38, 39]. The water from Little Black Pond has roughly the same noble gas pattern as the nine Greenland samples of Niu et al. [38] which are the “relative Ne depletion” samples. This is especially the case for KL-T2 (Fig 1C in Niu et al [38]) and similar to the pattern of sample 03 from the Athabasca Glacier (Fig 3B in Niu et al. [39]). Niu et al. [38] suggest that the Ne results are due to high equilibration elevation, between 2500 m and 3000 m, and an apparent equilibration temperature of 11°C. Because this temperature is inconsistent with expected temperatures over the surface of the glacier, they suggest a lack of water equilibration with surface conditions. They also propose that the concentrations of the heavier noble gases, and that of Xe in particular, are due to ice formation at higher altitudes rather than the apparently high equilibration temperature. These suggestions may apply here and can be tested by sampling the glaciers and alpine lakes directly.

As discussed above, the Axel Heiberg springs have been suggested as a model for permafrost flows on early Mars [7]. In this study, we have identified glacier melt and ice-covered lakes as the source of the water in these springs. Glaciers and perennially ice-covered lakes are thought to have been widespread on early Mars [42–44], and this analysis of the water sources of the Axel Heiberg springs may contribute to the quantitative understanding of liquid water outflows associated with these ice environments on Mars.

## Conclusions

We have measured the concentration of gases in bubbles and dissolved in the water emanating from Little Black Pond, a perennial spring located in the Canadian High Arctic in a region of thick, continuous permafrost. We have also determined the dissolved oxygen levels of the two lakes, Phantom Lake and Astro Lake, which are likely to be a source of water for this spring. From these data, we draw the following conclusions:

The partial pressures of each gas in the bubbles emerging from the spring are about 20% below the value expected if the bubbles are in equilibrium with the gas dissolved in the saline spring water.

The lack of dissolved oxygen is consistent with its consumption in the groundwater. The level of CO_2_ in the spring water is much too low to represent a significant sink of the O_2_ in the source waters. This implies that most of the consumption of O_2_ results in sulfate formation.

The results for ^3^He, ^4^He, and tritium, when taken together, imply that the residence time of the water in the spring is longer than 70 years and possibly many thousands of years.

The N_2_/Ar ratio and the Kr/Xe ratio are consistent with the source gas being an equal mix (fraction of lake water = 0.47±01) of gas from glacial bubbles and gas from air-saturated water–such as the water column of Phantom and Astro Lake.

Neon is 60% of its air value, which by comparison with similar Ne-depleted glacial meltwater in Greenland, may result from complex processes of ice formation on the source glaciers.
